# Role and Functions of Irisin: A Perspective on Recent Developments and Neurodegenerative Diseases

**DOI:** 10.3390/antiox14050554

**Published:** 2025-05-07

**Authors:** Aurelio Minuti, Ivana Raffaele, Michele Scuruchi, Maria Lui, Claudia Muscarà, Marco Calabrò

**Affiliations:** 1 IRCCS Centro Neurolesi “Bonino-Pulejo”, Via Provinciale Palermo, Contrada Casazza, 98124 Messina, Italy; aurelio.minuti@irccsme.it (A.M.); marco.calabro@irccsme.it (M.C.); 2 Department of Clinical and Experimental Medicine, University of Messina, 98124 Messina, Italy; mscuruchi@unime.it

**Keywords:** irisin, *FNDC5* gene, non-communicable diseases (NCDs), molecular pathways, reactive oxygen species (ROS)

## Abstract

Irisin is a peptide derived from fibronectin type III domain-containing protein 5 (FNDC5) and is primarily produced by muscle fibers under the regulation of peroxisome proliferator-activated receptor gamma coactivator 1-alpha (PGC1α) during exercise. Irisin has been the subject of extensive research due to its potential as a metabolic regulator and its antioxidant properties. Notably, it has been associated with protective actions within the brain. Despite growing interest, many questions remain regarding the molecular mechanisms underlying its effects. This review summarizes recent findings on irisin, highlighting its pleiotropic functions and the biological processes and molecular cascades involved in its action, with a particular focus on the central nervous system. Irisin plays a crucial role in neuron survival, differentiation, growth, and development, while also promoting mitochondrial homeostasis, regulating apoptosis, and facilitating autophagy—processes essential for normal neuronal function. Emerging evidence suggests that irisin may improve conditions associated with non-communicable neurological diseases, including Alzheimer’s disease, Parkinson’s disease, amyotrophic lateral sclerosis, frontotemporal dementia, and multiple sclerosis. Given its diverse benefits, irisin holds promise as a novel therapeutic agent for preventing and treating neurological diseases.

## 1. Introduction

Physical activity is widely recognized as a safe, accessible and effective intervention to enhance both metabolic and neurological health [1]. Its benefits have been extensively investigated in the medical field due to its potential as a non-pharmacological strategy for the prevention and management of several chronic diseases [2,3,4,5]. Elucidating the cellular mechanisms and molecular pathways underlying these beneficial effects is a growing area of scientific interest. Understanding these processes could inform the development of innovative therapeutic strategies, particularly for complex disorders such as neurodegenerative disorders [6,7].

In this context, myokines—bioactive molecules including cytokines, peptides, growth factors, and small proteins—have emerged as key mediators. Secreted by myocytes in response to muscular contractions, particularly during resistance and high-intensity exercise [8,9,10,11], myokines exert autocrine, paracrine, and endocrine effects across multiple target tissues, such as bone, adipose tissue, liver, and the brain [12]. Importantly, some myokines can cross the blood-brain barrier (BBB) and exert neuroprotective and neuromodulatory effects [12,13,14]. These molecules are central in the so-called muscle-brain axis (MBA), an integrative signaling pathway through which muscle activity modulates central nervous system (CNS) function [10,11,14]. Among the many myokines identified, brain-derived neurotrophic factor (BDNF), insulin-like growth factor-1 (IGF1), cathepsin B, vascular endothelial growth factor (VEGF), L-lactate, and FNDC5/irisin—play a prominent role in mediating exercise-induced benefits in the brain [10,11].

Among these, irisin has attracted considerable attention: irisin, named after Iris, the Greek goddess and messenger of Zeus, was discovered in 2012 by Boström et al. as a novel myokine produced and secreted by skeletal muscle in response to exercise, in both humans and mice [15]. Since then, this molecule has been extensively investigated, mainly for its potential as metabolic regulator and as a therapeutic agent for metabolic disorders [16,17,18], counteracting processes such as insulin resistance, inflammation, and cognitive decline [5]. These effects are particularly relevant for the prevention and management of neurodegenerative diseases such as Alzheimer’s Disease (AD) and Parkinson’s Disease (PD), as well as metabolic syndromes including type 2 diabetes and obesity [19,20]. Notably, increased irisin levels have been positively associated with improved cognitive function and memory performance [1,21,22]. Physical activity, particularly aerobic training, resistance exercise, and high-intensity workouts, has been shown to significantly elevate circulating irisin levels [23,24,25]. Due to these properties, irisin represents a potential therapeutic target for preventing and treating metabolic diseases, neurodegenerative pathologies and related conditions.

Despite the increasing interest in this myokine, the molecular mechanisms underlying irisin are not fully characterized, and its specific receptors have yet to be fully identified. Further research is therefore essential to elucidate its receptor binding, downstream signaling pathways, and functional role across various types of neurological NCDs. In this review, we will summarize the most recent irisin-related findings, and discuss its pleiotropic functions, providing a summary of the biological processes associated with irisin and the molecular cascades through which this molecule exerts its action. Finally, we will focus on its potential in the field of CNS and its related pathologies.

### Irisin: Biogenesis, Structure and Tissue Distribution

Irisin is the product of the proteolytic cleavage of fibronectin type III (FNIII) domain-containing protein 5 (FNDC5). The FDNC5 protein consists of 209 amino acid residues with an N-terminal signal sequence of 29 amino acids, an FNIII domain of 94 amino acids, a hydrophobic transmembrane domain of 19 amino acids, and a C-terminal region of 39 amino acids [26]. The C-terminal portion of FNDC5 is localized in the cytoplasm, while the N-terminal portion extends into the extracellular space. The proteolytic cleavage of FNDC5 produces irisin, a secreted peptide composed of approximately 112 amino acids, with a molecular weight of ~12 kDa [27]. Irisin structure has been identified by crystallographic experiments that highlighted its homodimeric structure composed by two beta sheets linked in antiparallel form, with an N-terminal domain structurally similar to FNIII and a flexible C-terminal tail [27,28,29]. Distinct from any previously solved FNIII structure, irisin forms a continuous inter-subunit β-sheet dimer. Dimerization appears to be an essential mechanism for irisin’s autocrine and paracrine signaling [27].

This molecule was initially identified as a peptide secreted by skeletal muscle. In addition to this origin, it was later discovered that white adipocytes are also capable of synthesizing irisin. As a result, irisin is classified not only as a myokine but also as an adipokine, leading to its designation as an adipomyokine [30].

Irisin is now known to be produced in several other tissues, including cardiac muscle, skin (sebaceous glands), testes, pancreas (islets of Langerhans, serous acinar cells, and cells of the intralobular and interlobular ducts), mammary gland, spleen, stomach (gastric parietal cells and cells of the muscular layer), and liver (hepatocytes, Kupffer cells, and sinusoidal endothelial cells) [31,32]. Of particular interest, histochemical studies have revealed the presence of irisin in the brain, both in neurons and neuroglia [31,33,34]. This broad tissue distribution suggests that irisin may have diverse physiological roles; nevertheless, skeletal muscle remains the primary source of this myokine, especially in response to physical exercise. We will therefore continue to refer to irisin as a myokine.

## 2. Irisin’s Function and Systemic Effects

Originally discussed in the field of exercise physiology [15,35], irisin has gained increasing attention in several scientific areas due to its potential pleiotropic effects [16,17,18]. Elevated in healthy states and reduced in diseases, irisin mediates numerous beneficial effects across various tissues and organs, including skeletal muscle, adipose tissue, bone, liver, and the CNS [29,36,37,38].

Initially, the role of irisin in humans has raised a lot of critics and discussions due to the presence of an “anomalous” ATA start codon in the human *FNDC5* gene. In vitro evaluations of its expression have resulted in a very limited production of *FNDC5* RNA, suggesting that the encoded protein may not play a significant role [39]. This view was further boosted due to technical difficulties in detecting irisin protein in the bloodstream [40,41]. However, recent studies have shifted this perspective, demonstrating that *FNDC5* is expressed in humans and that its transcription is likely controlled by an ATG start codon located on the 5′ upstream of the originally identified start codon [39].

**Figure 1:**
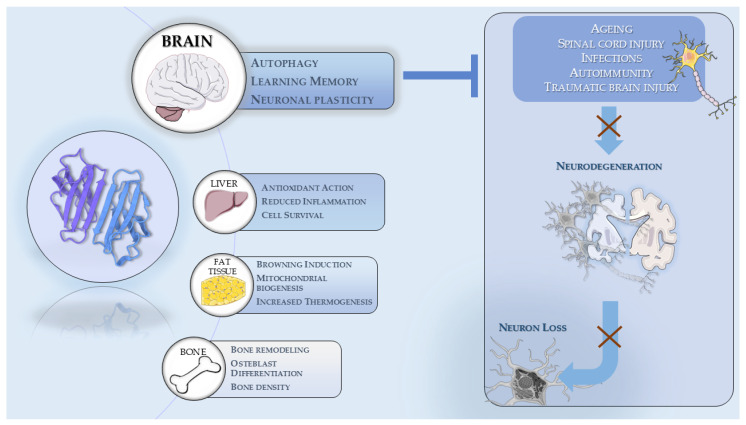
The main functions of irisin are summarized in the various body districts, with a particular emphasis on CNS. The action of irisin counteract neurodegeneration and neuron loss that can be triggered by several events (i.e., Ageing, infections, Injuries). The image was created using the image bank of Servier Medical Art (available online: http://smart.servier.com/), licensed under a Creative Commons Attribution 4.0 International License (available online: https://creativecommons.org/licenses/by/4.0/, accessed on 27 March 2025).

As previously stated, irisin is produced by the cleavage of the transmembrane protein encoded by *FNDC5*, which is subsequently released into the bloodstream [42]. Once in the bloodstream, irisin reaches several body districts where it triggers a wide range of biological processes. In this section, we discuss the functions of this myokine across different body tissues and then outline its key protective effects at the systemic level. Figure 1 summarizes the main functions of irisin.

### 2.1. Impact on Muscle, Bone and Other Peripheral Tissues

Irisin and its precursor, FNDC5, play critical roles in muscles, and have been extensively studied in relation to exercise [43]. Muscle tissue not only serves as the main source of irisin [44], but it is also considered one of its main targets, where irisin primarily exerts autocrine/paracrine effects [44]. Here, FNDC5 levels are correlated to baseline muscle fiber composition abundance [45]. In vitro experiments suggested that irisin enhances myogenesis, primarily by promoting myoblasts’ fusion [46]. This action protects against muscle atrophy and enhances muscle regeneration [47,48,49]. Irisin also plays a role in muscle metabolism as it augments amino acids, fatty acids and glucose uptake into muscle cells [50,51,52] and concomitantly upregulates glycolysis and lipid β-oxidation, and downregulates glycogenolysis processes [51,53].

Focusing on bone tissue, irisin has been shown to play important roles in bone remodeling and the overall skeletal health [54,55,56,57]. Several studies have shown that high irisin levels enhance osteoblast differentiation, attenuate osteoclast activity, and increase bone density. These processes are essential for bone maintenance and integrity, as well as for the prevention of negative effects related to aging and/or other conditions, such as osteoporosis [57,58,59,60].

Another well-characterized role of irisin is the induction of “browning” in white adipose tissue (the trans-differentiation of white adipocytes into brown-like fat cells) [15,61,62,63]. Irisin promotes browning by enhancing mitochondrial biogenesis and increasing energy-to-thermal conversion by elevating the levels of uncoupling protein-1 (UCP1) [61] and other thermogenic proteins [64,65,66], along with other mitochondrial-related biological processes [67,68,69]. These processes contribute to increased thermogenesis and have potential implications for the treatment of different metabolic syndromes such as obesity and diabetes [15,64,70,71,72]. Conversely, in white preadipocytes, irisin treatment does not induce browning but inhibits adipogenesis, thereby reducing the formation of new adipocytes [62,73].

### 2.2. Irisin’s Neuroprotective Role and CNS Relevance

Focusing on potential irisin benefits in the CNS, it was observed that, irisin can cross the BBB, suggesting that systemic production of this myokine may have relevant effects on the brain [74]. Additionally, it has been observed that neurons express FNDC5 and several brain areas produce irisin locally. This is of particular interest, since the correct function of the FNDC5/irisin network seems to be necessary for the optimal functioning of the CNS, and mutations in the *FNDC5* gene have been associated with neurodegeneration and pathological conditions, such as AD [75]. In this context, it should also be noted that irisin is one of the few known stimuli capable of inducing de novo neurogenesis [42,76,77], which also attenuates neuronal damage and synaptic loss [78,79,80,81,82,83]. Indeed, in vitro and in vivo studies have shown that irisin enhances the differentiation of neural progenitor cells and increases the expression of neurotrophic factors such as BDNF [84,85,86,87]. In this setting, the enhanced BDNF signaling triggered by irisin promotes synaptic plasticity, dendritic spine formation, and, ultimately, improves cognitive function [88,89]. Irisin also promotes anti-inflammatory, antioxidant, and other neuroprotective effects, ranging from apoptosis mitigation to immune response and cytokine secretion management [42,78,80,83,85,86,87,90]. Indeed, in various models of neural injury, irisin administration reduces pro-inflammatory cytokines expression [80,83,91,92]. Simultaneously, irisin activates antioxidant pathways, that protect neurons from oxidative damage [85,93,94]. This dual action is particularly relevant in neurodegenerative diseases, where chronic inflammation and oxidative stress contribute to progressive neuronal loss.

Irisin’s metabolic signaling may also indirectly influence CNS bioenergetics via lactate shuttling. In isolated cell systems, this myokine stimulates glycolytic flux and lactate secretion, primarily through mild mitochondrial uncoupling, and activation of AMP-activated protein kinase (AMPK), Mitogen-Activated Protein Kinase 1/3 (MAPK1/3 or ERK1/2) and Mitogen-Activated Protein Kinase 14 (MAPK14) pathways [95,96]. These in vitro observations suggest that irisin could elevate overall lactate levels, a property of high relevance for neuronal health, since neurons are avid utilizers of lactate for energy. Indeed, neurons express high levels of monocarboxylate transporter 2 (MCT2) to import L-lactate and convert it to pyruvate via lactate dehydrogenase (LDH); pyruvate enters the tricarboxylic acid cycle (TCA) and the electron transport chain, ultimately sustaining ATP production, buffering redox status, and reducing oxidative stress in neuronal populations [97,98]. Furthermore, in CNS lactate not only functions as a metabolic substrate to provide energy but can also function as a signaling molecule to modulate cellular functions, through protein lactylation, under pathophysiological conditions [99].

Notably, increasing CNS lactate availability has been shown to slow neurodegenerative progression. For example, lactate administration in murine models rescues long-term potentiation and learning deficits and reduces amyloid-beta (Aβ) burden in AD models [100,101], and it enhances spatial memory in healthy mice [102]. However, excessive lactate can be detrimental: elevated levels exacerbate PD symptoms [103], and may trigger neuroinflammation—particularly under mitochondrial stress, leading to pro-degenerative effects [99]. Interestingly, such detrimental effects may be countered by irisin action through other biological cascades. As such, we can hypothesize that lactate increase by irisin administration could elicit lactate’s beneficial effects, while mitigating the negative ones via irisin’s activation of anti-inflammatory and antioxidant pathways.

Despite the potential of irisin in inducing lactate in some cell models and although exercise-induced irisin and lactate rise in parallel, causality between irisin and lactate increase in vivo remains to be established. Furthermore, the lactate production-inducing effects of irisin in CNS cells, including neurons and astrocytes, are yet to be investigated. Consequently, any proposed benefits of irisin-mediated lactate elevation should be further validated and carefully interpreted since, as previously described, excessive lactate production triggers negative effects.

As it will be better detailed later in the text, irisin also plays a crucial role in ameliorating mitochondrial dysfunction. This function is particularly relevant in neurons, where this molecule promotes the clearance of damaged mitochondria through mitophagy [57,74]. By enhancing cellular resilience under stress, irisin helps maintain energy homeostasis and promotes neuron survival. Furthermore, irisin is able to preserve the CNS microenvironment by exerting a protective and stabilizing effect on the BBB [79,81,93]. Specifically, it has been demonstrated that this myokine enhances the expressions of several proteins, including occludin, claudin 5, and tight junction protein 1, which are part of the tight junctions in the BBB [85]. This effect results in an increased stability of such junctions and, subsequently, in a reduced BBB permeability [104,105,106].

### 2.3. Protective Effects of Irisin: Apoptosis

Irisin modulates several apoptotic and regulated cell death pathways, thereby contributing to cellular protection and tissue homeostasis [42,78,81,107]. Specifically, irisin has been shown to reduce classical apoptosis by decreasing the expression of pro-apoptotic proteins, such as BCL2 Associated X, Apoptosis Regulator (BAX) and cleaved caspase-3, while upregulating anti-apoptotic proteins like BCL2 Apoptosis Regulator (BCL2) [81,86,108], which enhances cell survival in multiple cell types [49,109]. This myokine also influences alternative apoptosis-related mechanisms; specifically, it can attenuate pyroptosis—a highly inflammatory form of programmed cell death mediated by the NLR Family Pyrin Domain Containing 3 (NLRP3) inflammasome—by inhibiting key components of the pyroptotic cascade [110,111,112]. Irisin also can counter ferroptosis, which is characterized by iron-dependent lipid peroxidation: in this context, irisin seemingly acts through activation of the NRF2/GPX4 signaling axis [93,113], and/or by inhibiting EIF2A/ATF4/DDIT3 pathway [114] to boost cell survival [114,115]. Although evidence for irisin’s effects on necroptosis is still not fully characterized, some recent findings suggest that this myokine may also interfere with necroptotic signaling pathways, further contributing to its cell-protective profile [116].

### 2.4. Protective Effects of Irisin: Autophagy

Autophagy is a crucial process for maintaining cellular homeostasis, which involves lysosomal degradation of damaged proteins and organelles, and plays a key role in overall cellular stability [117]. It is classified into three types: macroautophagy, microautophagy, and chaperone-mediated autophagy (CMA) [118]. Macroautophagy, the most prevalent form, involves autophagosomes that enclose cellular waste and merge with lysosomes for recycling and degradation [119]. Microautophagy involves the direct uptake of cytoplasmatic material through the invagination of lysosomal membrane [120]. CMA targets specific proteins identified by chaperones, which are subsequently transported to lysosomes for degradation [121]. Irisin has been shown to play a pivotal role in regulating autophagy, particularly macroautophagy [122]. Notably, autophagy is often impaired in many neurodegenerative disorders [123,124], and several studies have demonstrated that irisin can restore this function [125]. For example, irisin administration in aging liver cells after ischemia-reperfusion enhances autophagic activity by inhibiting Mitogen-Activated Protein Kinase 8 (MAPK8) phosphorylation, thus increasing telomerase activity and supporting tissue repair [126].

### 2.5. Protective Effects of Irisin: Mitochondrial Homeostasis

In relation to both apoptosis and autophagy processes, it should be noted that irisin exerts part of its neuroprotective functions by modulating mitochondria dynamics [127]. Mitochondrial health depends on a dynamic equilibrium between fission, fusion, and mitophagy, with mitophagy playing a key role in removing damaged mitochondria to prevent oxidative stress and cell damage. In this context, it has been observed that irisin is able to promote fusion by upregulating mitofusin 2 (MFN2) and OPA1 Mitochondrial Dynamin Like GTPase (OPA1) expression, which are proteins essential for outer- and inner-membrane merging [68]. Simultaneously, irisin can inhibit excessive fission by preventing Dynamin 1 Like (DNM1L) phosphorylation through the normalization of JNK-LATS2 signaling pathway [128].

Irisin also promotes elective clearance of damaged mitochondria through the PTEN Induced Kinase 1/Parkin (PINK1/PRKN) pathway [69]. PINK1, PRKN, Microtubule Associated Protein 1 Light Chain 3 Beta (MAP1LC3B) and Sequestosome 1 (SQSTM1) are key proteins regulating mitophagy [129]. Irisin elevation has been shown to amplify this pathway in murine models, resulting in a lowered ROS burden and reduced apoptotic signaling activation [69]. OPA1 upregulation by irisin has also been associated with increased mitophagy [130,131,132].

Additionally, upon binding to integrin αVβ5 receptors, irisin activates PI3K/AKT and MAPK1/3 signaling, leading to transcriptional upregulation of *PGC1α* gene and downstream nuclear–mitochondrial factors (TFAM, NRF1 and NRF2) [133], which collectively enhance mtDNA replication and transcription to boost new mitochondrial biogenesis [134].

Ultimately, these effects are particularly relevant in neurodegenerative disorders, as Mitochondria play a crucial role in regulating apoptosis by releasing pro-apoptotic factors like cytochrome c, and excessive apoptotic activity contributes to neuronal death in these diseases [135]. Additionally, mitochondria are involved in antioxidant defense by controlling reactive oxygen species (ROS) levels, which can cause cellular damage [136,137]. By averting mitochondrial collapse/fission and enhancing their function, irisin could be a promising element to counteract increased inflammation and cell death typically observed in neurodegenerative processes.

### 2.6. Protective Effects of Irisin: Anti-Inflammatory and Antioxidant Actions

In addition to its established roles in metabolism and cell survival, the potential of irisin to counteract inflammatory states has also been extensively investigated [42,138,139,140]. This myokine exerts its anti-inflammatory effects by modulating several signaling pathways [139,140]. For example, irisin has been shown to inhibit the TLR4/MYD88/NF-κB pathway, resulting in a reduction of the production of pro-inflammatory cytokines such as Tumor Necrosis Factor-alpha (TNFα), Interleukin 6 (IL6), and Interleukin 1 beta (IL1β), overall mitigating chronic inflammation [138,141,142,143,144]. Additionally, as previously described, FNDC5/irisin inhibits NLRP3 inflammasome activation [110,111,112], significantly down-regulates the PPARγ/PGC1α/FNDC5 axis, and reduces inflammatory state in an in vitro model of inflammation (pancreatitis) [145]. Concurrently, this myokine also promotes the activation of antioxidant pathways, mainly through the activation of NFE2 Like BZIP Transcription Factor 2 (NRF2 or NFE2L2) [85,93,142], thereby enhancing cellular defense mechanisms against oxidative stress [108]. Irisin also is able to reduce oxidative stress by countering the production of ROS [85,93].

Interestingly, irisin’s anti-inflammatory and antioxidant properties have sparked interest in its potential neuroprotective function [74], as inflammation and oxidative stress are a common denominator in multiple neurodegenerative diseases. Abnormal ROS production leads to neuronal dysfunction, thereby accelerating the progression of the disease [146]. In this context, irisin can promote mitochondrial biogenesis and increase the expression of antioxidant enzymes. For instance, exogenous irisin treatment has been shown to enhance the expression of key mitochondrial regulators, such as PGC1α and Transcription Factor A, Mitochondrial (TFAM) [146], which in turn increases the levels of antioxidant enzymes including superoxide dismutase (SOD) and catalase [147].

Irisin also promotes the transition of microglia from a pro-inflammatory to an anti-inflammatory state by activating the AMPK pathway, which in turn reduces *IL1β* expression [42]. Further, it has been observed that irisin can effectively inhibit microglial activation and monocyte infiltration by activating MAPK1/3 and AKT Serine/Threonine Kinase (AKT) signaling pathways in a cerebral ischemia model [82]. This leads to a significant reduction in the expression of *TNFα* and *IL6* mRNA, as well as a decrease in oxidative stress markers [82]. In a lipopolysaccharide-induced neuro-injury model, irisin activates the NRF2/HMOX1 signaling pathway, effectively reducing the expression of inducible nitric oxide synthase (*iNOS*) and cyclooxygenase-2 (*MT-CO2*), which are crucial enzymes involved in inflammation and oxidative stress [148].

Additionally, irisin suppresses several important signaling pathways activated by lipopolysaccharide, including the phosphorylation of MAPK14, MAPK1/3, MAPK8, and interferon regulatory factor 3 (IRF3), as well as the degradation and phosphorylation of Inhibitor Of NF-κB (IκB), a key regulator of NF-κB [148].

Irisin also exerts anti-inflammatory effects by enhancing the expression of *BDNF*. BDNF binds to its high-affinity receptor, Neurotrophic Receptor Tyrosine Kinase 2 (NTRK2), which in turn activates the cAMP response element-binding protein (CREB) signaling pathway. This process effectively reduces NF-κB activation, further suppressing the production of pro-inflammatory cytokines like IL6 and IL1β, thereby significantly easing the inflammatory condition in neural tissue [149].

## 3. Molecular Cascades and Receptor Interactions

**Figure 2:**
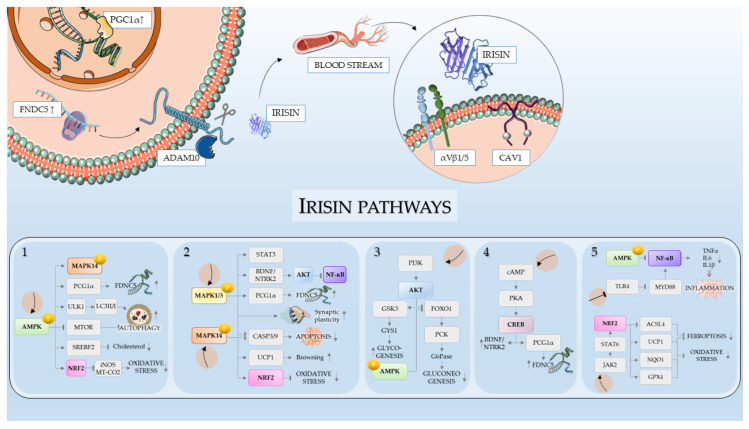
Schematic representation of the steps involved in irisin production and the molecular cascades underlying its biological functions. Each signaling pathway was schematized and simplified to improve clarity and readability. Interactions between different pathways are highlighted using a color-coding system (e.g., AMPK activates NRF2 in pathway 1, and NRF2 activation continues in pathway 5). The numbering of the pathways corresponds to the paragraphs in Section 3. Arrows at the right-hand side of proteins and pathways indicate if their expression is increased (↑) or decreased (↓) by irisin. The figure was created using the image bank of Servier Medical Art (available online: http://smart.servier.com/), licensed under a Creative Commons Attribution 4.0 International License (available online: https://creativecommons.org/licenses/by/4.0/, accessed on 27 March 2025). ACSL4: Acyl-CoA Synthetase Long-Chain Family Member 4; ADAM10: Disintegrin and metalloproteinase domain-containing protein 10; AMPK: AMP-activated protein kinase; αV/β5: Alpha-v beta-5 receptor; BDNF: Brain-derived neurotrophic factor; CASP3/9: Caspase 3/9; cAMP: Cyclic adenosine monophosphate; CAV1: Caveolin-1; CREB: cAMP response element-binding protein; FNDC5: Fibronectin type III domain-containing protein 5; FOXO1: Forkhead box protein O1; G6Pase: Glucose-6-phosphatase; GPX4: Glutathione peroxidase 4; GSK-3: Glycogen synthase kinase-3; GYS1: Glycogen synthase; MAPK: Mitogen-activated protein kinase; MYD88: Myeloid differentiation primary response 88; NF-κB: Nuclear factor-κB; NQO1: NAD(P)H Quinone Dehydrogenase 1; NRF2: Nuclear factor erythroid 2-related factor 2; NTRK2: Tropomyosin receptor kinase B; PCK: Phosphoenolpyruvate carboxykinase; PI3K: Phosphoinositide 3-kinase; PGC1α: Peroxisome proliferator-activated receptor-gamma coactivator; PKA: Protein Kinase cAMP-Activated; STAT3: Signal transducer and activator of transcription 3; UCP1: Uncoupling protein 1.

Although several studies have highlighted the pleiotropic effects of irisin, which we summarized in the previous section, a detailed characterization of the molecular cascades activated or inhibited by this myokine is still lacking. In this section, we focus on the main genes, proteins and signaling pathways involved with irisin regulation and its effects. Figure 2 reports the main molecular pathways involved in the irisin signal.

### 3.1. FNDC5 Expression and Cleavage

The initial step in irisin biology is the expression of its precursor, FNDC5, in several tissues [35,150,151], followed by its proteolytic cleavage, which releases irisin [15]. As previously reported, multiple studies on the *FNDC5* gene have demonstrated that its expression is regulated by PGC1α [15,152]. PGC1α is a transcriptional coactivator which regulates mitochondrial biogenesis and energy metabolism in response to various physiological stimuli. Its expression is promoted by several factors, mainly associated with exercise [153,154] and cold exposure [155]; and is downregulated by chronic inflammation and oxidative stress [156]. Transforming growth factor-β (TGFβ) and its effector protein SMAD Family Member 3 (SMAD3) have also been proven to negatively regulate the expression of *PGC1α* [157]. Consequently, *FNDC5* expression is also associated with the mentioned stimuli, as confirmed by several studies, especially for those related to physical exercise [15,158]. Notably, the *FNDC5* increased expression is not directly proportional to irisin production [127]. In fact, several observations have highlighted that increases in *FDNC5* expression and irisin levels occur at different times during and after exercise stimuli [44,159,160]. This evidence leads to the hypothesis that the key process for irisin production may be the activation of FNDC5 cleavage, rather than its upregulation [161,162,163]. Unfortunately, the molecular mechanisms underlying FNDC5 cleavage control are still not fully understood, as the protease responsible for FDNC5 cleavage is yet to be identified. Nevertheless, recent evidence suggests that members of the ADAM (a disintegrin and metalloproteinase) family, particularly ADAM Metallopeptidase Domain 10 (ADAM10), may be involved in this process [44].

To add to the complexity of FDNC5/irisin expression dynamics, some observations in the literature support the existence of alternative forms of FNDC5, either uncleaved or partially cleaved [164]. These forms may also be biologically relevant and could account for some of the observed variability in irisin detection and functions, including those related to neuronal development [165,166,167].

### 3.2. Receptor Identification: The Role of Integrins

An important breakthrough in understanding the molecular events behind irisin’s mechanism of action came with the identification of its potential receptor [64]. Several studies have demonstrated that irisin binds with high affinity to the integrin αV/β5 receptor complex [168,169]. Additionally, some studies have indicated that the affinity of irisin with this receptor can be further enhanced by the presence of Heat Shock Protein 90 Alpha (HSP90α), a molecular chaperone that stabilizes protein interactions [170].

Integrins are transmembrane receptors that mediate interactions between cells and the extracellular matrix, transducing signals that influence cell survival, proliferation, and differentiation. Notably, αV/β5 integrin is expressed in various tissues and cell types, including adipose tissue [30], bone [64], brain [42], and intestine [171]. The binding of irisin to this receptor has been implicated in several downstream signaling cascades that contribute to its diverse biological effects.

For instance, in osteocytes and adipocytes, irisin binding to αV/β5 integrin activates the focal adhesion kinase (FAK) pathway, which in turn influences cytoskeletal dynamics and cell survival [169]. Similarly, in muscle cells, irisin binding to this integrin has been linked to the activation of AMPK, a key regulator involved in inflammation management, energy metabolism, and mitochondrial biogenesis [49,52,172,173,174,175,176].

Beyond αV/β5, evidence suggests that other integrin subunits, such as β1 integrin, may also play a role in irisin signaling in specific cell types [169]. However, the potential presence of other receptors for irisin remains a subject of ongoing debate [177,178]. For example, a study by Tao et al. discussed the interaction of FNDC5/irisin with caveolin 1 (CAV1) in osteoblasts [54], showing that this interaction plays a crucial role in promoting cell survival [54]. The identification of irisin receptors is crucial for understanding irisin-activated signaling cascades and may also provide insights into the potential tissue-specific actions of this myokine. Interestingly, it should also be noted that the FNIII domain present in the FNDC5 protein is a domain commonly contained in receptor proteins. This similarity may support a possible role for the uncleaved form as a receptor whose ligand has yet to be identified [35].

### 3.3. Downstream Signaling Pathways

As previously introduced, once irisin binds to its receptor, several intracellular signaling cascades can be triggered. In this section, we will focus on the main molecular cascades involved in irisin signal transduction that are responsible for its biological effects.

#### 3.3.1. AMPK Pathway

Activation of AMPK is one of the most described effects of irisin [42,179,180]. This pathway is essential for maintaining energy homeostasis and is primarily activated directly by irisin through its interaction with integrin αVβ5 [42], and CAV1 in some cell types [54]. AMPK activation enhances glucose uptake through the upregulation of Solute Carrier Family 2 (Facilitated Glucose Transporter), Member 4 (SLC2A4 or GLUT4) [52,174,176] as well as mitochondrial biogenesis and autophagy by activation of Unc-51 Like Autophagy Activating Kinase 1 (ULK1) signaling pathway [59,180,181] and the AMPK/MTOR axis [182]. It also reduces gluconeogenesis by reducing Phosphoenolpyruvate Carboxykinase (PCK) and Glucose-6-Phosphatase (G6Pase) [51]; cholesterol synthesis by inhibiting Sterol Regulatory Element Binding Transcription Factor 2 (SREBF2) [183]; inflammation by reducing NF-κB levels and *IL1β* expression [42,173,184]; and oxidative stress by down-regulating the expression of *iNOS* and *MT-CO2* genes through NRF2 [148]. The AMPK pathway signal, activated by irisin, is also documented to protect from apoptosis in some cell types [171]. Research has also demonstrated that irisin promotes macroautophagy by activating the AMPK/MTOR signaling pathway [185].

Interestingly, AMPK activation seems to be able to regulate, possibly through a feedback mechanism, PGC1α and, in turn, *FNDC5* gene expression [173,181,186]. Based on these studies, it seems that AMPK can act as both an upstream and downstream mediator of the PGC1α/FNDC5 axis [173,181,186]. A detailed discussion on the correlation between AMPK and irisin has been recently proposed by Laurindo et al. [187].

#### 3.3.2. MAPK/ERK Pathway

The mitogen-activated protein kinase (MAPK) cascade, particularly the MAPK1/3 pathway, is modulated by irisin in several cell types. The MAPK pathway is a crucial regulator of cellular processes such as proliferation, differentiation, metabolism, and apoptosis [188]. Among the multiple MAPKs regulated by irisin, MAPK14 is involved in glucose metabolism [52,189], adipocyte browning [62] and proliferation in some bone-related cell types [190,191,192]. MAPK1/3 is also involved in the browning of adipose tissue. Indeed, inhibition of both *MAPK14* and *MAPK1/3* genes’ expression blocks the induction of *UCP1* expression by irisin [193]. In neurons, MAPK1/3 activation is particularly important as it contributes to synaptic plasticity, neuronal survival, and the regulation of neuroprotective gene expression [175,194]. Through this pathway, irisin also mediates neuroinflammation [82]. Interestingly, MAPKs can enhance the transcription of the *PGC1α* gene, and in turn increase *FNDC5* expression [187].

#### 3.3.3. PI3K/AKT Pathway

Irisin has also been shown to engage the PI3K/AKT signaling pathway [175]. This cascade is critical for promoting cell survival and regulating metabolic functions: Phosphatidylinositol-4,5-Bisphosphate 3-Kinase (PI3K) activation triggers Glycogen Synthase Kinase 3 (GSK3) which results in the reduction of gluconeogenesis and the concomitant induction of glycogen synthesis [195,196]. Additionally, PI3K/AKT, through the dephosphorylation of Insulin Receptor Substrate 1 (IRS1) and Insulin Receptor Substrate 2 (IRS2) [197], also counteracts the development of insulin resistance [198]. PI3K/AKT also mitigates inflammation, by reducing *TNFα* and *IL6* expression [199,200]. Some studies have highlighted that the potential anti-metastatic effects of irisin depend in part on its regulation of the PI3K/AKT pathway [201], although other studies provide contradictory evidence [202]. In the context of neurodegeneration, PI3K/AKT activation may help protect neurons [203]. It has also been observed that this pathway may have an antidepressant action [204], which could potentially be triggered by irisin.

#### 3.3.4. cAMP/PKA/CREB Pathways

Experimental data have highlighted that irisin can stimulate the cAMP/PKA/CREB pathway [205]. Through this pathway, irisin exerts protective effects, especially in the CNS [205,206]. Indeed, CREB has a well-documented role in neuronal plasticity and long-term memory formation in the brain [207,208]. Irisin, by activating CREB, positively influences memory formation and cognitive function [205,206]. Interestingly, CREB activation enhances FNDC5 synthesis, whereas its inactivation suppresses PGC1α-induced *FNDC5* expression [194,209].

#### 3.3.5. NRF2 and NF-κB Pathways

Through the activation of the NRF2 pathway and inhibition of NF-κB signaling, irisin reduces the production of inflammatory cytokines, thus mitigating inflammatory responses both peripherally and centrally. Irisin promotes the expression, phosphorylation and nuclear translocation of NRF2, activating its downstream signaling [210]. This effect is obtained through the previously described pathways. Additionally, irisin interacts with integrin αVβ5, promoting Janus Kinase 2 (JAK2) phosphorylation, which in turn activates the Signal Transducer and Activator of Transcription 6 (STAT6). This activation enhances the transcription of NRF2 and promotes its translocation to the nucleus [210]. The cascade of events downstream of NRF2 translocation leads to the upregulation of antioxidant genes such as heme oxygenase-1 (*HMOX1*), NAD(P)H Quinone Dehydrogenase 1 (*NQO1*), and Glutathione Peroxidase 4 (*GPX4*), enhancing cellular defense against oxidative stress. Additionally, NRF2 activation of GPX4, in concert with the inhibition of Acyl-CoA Synthetase Long-Chain Family Member 4 (*ACSL4*) expression, prevent ferroptosis [93,115]. Concurrently, irisin inhibits NF-κB by preventing the phosphorylation and degradation of IκB, blocking NF-κB nuclear translocation, and reducing the transcription of pro-inflammatory cytokines such as TNFα and IL6. The inhibition of NF-κB seems to be related to the action of this myokine on the expression of *TLR4* and *MYD88* genes: irisin-induced downregulation of their expression leads to decreased activation of NF-κB [21,80]. Both these mechanisms play a crucial role in neuroprotection.

#### 3.3.6. Other Pathways

The Notch signaling pathway plays a key role in cell differentiation and neuronal function [211]. Studies have reported that irisin enhances the expression of Notch proteins and promotes their cleavage into the active Notch intracellular domain (NICD), thereby facilitating gliogenesis, neuronal differentiation, and microglial activation [78]. Blocking Notch cleavage reverses the neuroprotective effects of irisin, confirming that the action of this myokine is indeed linked to the Notch pathway [78]. In the context of microglia, irisin could promote M2 microglia polarization, and this effect is mediated through the hypoxia-inducible factor-1 subunit α (HIF1α) pathway [212]. The activation of Peroxisome Proliferator Activated Receptor Gamma (PPARγ) signaling cascade by irisin also appears to promote M2 induction [210]. Additionally, irisin inhibits microglial senescence by modulating *TFAM* expression. This effect appears to be mediated through the SIRT1/PGC1α signaling pathway [213].

Of particular interest, irisin promotes the production of BDNF [214,215]. BDNF is essential for many aspects of brain development including neuron survival and differentiation, as well as synaptogenesis and plasticity [216,217], which in turn are critical for memory and learning [218]. The ability of irisin to modulate BDNF production makes this myokine particularly relevant in the field of CNS pathologies, especially neurodegenerative disorders, due to its potential therapeutic effects and cognitive benefits [219].

In muscle, irisin also activates IGF1 signaling, promoting the uptake of glucose and amino acids in muscle cells [50].

Irisin also acts as a Signal Transducer and Activator of Transcription 3 (STAT3) regulator to reduce inflammation (in concert with MAPK14 and NF-κB) [83]. The regulation of STAT3/SNAI1 signaling pathway has also been demonstrated to have anti-metastatic effects, as it reverses the IL6 induced epithelial–mesenchymal transition [220]. Finally, irisin regulates autophagy through the WNT signaling pathway [221].

Collectively, the ability of irisin to modulate multiple signaling pathways highlights the pleiotropic nature of this myokine. Furthermore, it should be noted that the regulatory effects of irisin may also depend on its ability to control the expression of certain non-coding RNAs (ncRNAs), through which this myokine modulates gene expression, as observed in recent studies [222,223,224,225,226].

## 4. Irisin and Neurodegenerative Diseases

The CNS is a crucial component of the nervous system, responsible for processing and integrating internal and external information, and generating coordinated responses to these stimuli [227]. The CNS is made up of two interconnected organs, the brain and the spinal cord, that are protected by three layers of meninges and are enclosed within the bony structures [227]. The CNS plays a crucial role in regulating immune responses and inflammation [228]. Neuroinflammation plays a critical role in many neurological disorders and has emerged as a key target for therapeutic interventions. These disorders affect neurons and other components of the nervous system, resulting in a broad spectrum of symptoms [228].

In recent years, attention has turned toward the role of irisin in the CNS and its potential as a biomarker for neurodegenerative and neuroinflammatory diseases [229]. This shift is largely attributed to the decline in infectious diseases and the growing aging population, which has led to an increased prevalence of non-communicable diseases (NCDs), such as neurodegenerative disorders [230].

Although FNDC5 and irisin are expressed in several brain regions, including the hippocampus, cortex, and cerebrospinal fluid, the irisin detected in the CNS likely originates from peripheral secretion and local neural production.

Interestingly, irisin has shown potential in modulating the CNS by mitigating neuronal loss, cognitive decline, synaptic dysfunction, and neuroinflammation commonly associated with neurodegenerative diseases, such as AD and PD. As previously discussed, these effects are mainly obtained through the upregulation of *BDNF* and the anti-inflammatory properties of irisin, which support neuron survival, synaptic plasticity. Multiple studies support the existence of a signaling axis involving PGC1α, FNDC5, and BDNF [231]; and BDNF is known to play a key role in regulating synaptic transmission and long-term potentiation in the hippocampus and other areas of the brain [232]. Overall, irisin effects counteract the pathological conditions commonly present in multiple neurodegenerative diseases (e.g., neuro-inflammation, neurodegeneration) [74,231]. In AD, for instance, cerebrospinal fluid levels of irisin correlate with AD biomarkers and clinical dementia scores [233,234], and animal models show that higher irisin levels are associated with reduced amyloid pathology and improved memory [94,205,235]. Similarly, in PD, irisin protects dopaminergic neurons by reducing oxidative stress, α-synuclein (α-syn) pathology, and pro-inflammatory signaling [42,107,125,236]. Additionally, serum irisin levels have been linked to cognitive status in vascular dementia [237].

Additionally, as aging reduces brain insulin levels and contributes to insulin resistance, a significant risk factor for neurodegeneration, irisin’s capacity to counteract this process [197,198] becomes particularly relevant.

The role of irisin was also recently investigated in the context of postoperative cognitive dysfunction (POCD) since it represents a multifactorial neurodegenerative disease characterized by a decline of patient’s cognitive ability in the days after surgery and is prevalent in 1% of elderly patients [38]. Irisin preoperative deficiency in individuals over 70 years old was associated with an increased risk of developing dementia after surgery. Irisin prevents POCD-like behavior by counteracting the surgery-induced neuroinflammatory responses. It has been observed that in vivo administration of irisin is able to prevent memory deficits and cognitive impairments in behavioral tasks on a mouse model of neuroinflammation. This effect is likely elicited by the activation by irisin of the STAT6 signaling pathway in microglia, which promotes the anti-inflammatory phenotype [38].

Although many aspects of the neuroprotective properties of irisin remain unclear, in this section, we focused on the function and therapeutic potential of irisin in neurodegenerative diseases. We believe that this is of great importance because of “aging” and “increasingly sedentary” population, which are both relevant factors that increase the risk of neurodegenerative conditions.

### 4.1. Irisin and Alzheimer’s Disease

AD is a neurodegenerative disorder characterized by memory loss, dementia, and progressive cognitive decline, which severely impacts patients and their families [238]. AD is characterized by Aβ deposition, tau hyperphosphorylation, neuroinflammation, mitochondrial dysfunction, synaptic loss, and reduced nerve growth factor (NGF) levels [238,239]. Its progression involves hippocampal atrophy, microglial activation, brain energy dysfunction, and neuronal apoptosis [239]. Several modifiable risk factors have been identified, including diabetes, hypertension, obesity, smoking, depression, and physical inactivity, with exercise emerging as the most effective preventive strategy due to its impact on multiple pathways implicated in AD [240,241]. Exercise activates the PGC1α/FNDC5/irisin pathway, helping to prevent age-related cognitive decline [242,243]. Notably, endurance athletes exhibit higher irisin levels, which positively correlate with cognitive performance [244]. Conversely, genetic deletion of FNDC5 accelerates aging and cognitive dysfunction in mice, effects that can be alleviated by increasing circulating irisin levels [245].

Knockout (FNDC5/irisin KO) mice exhibit cognitive deficits in object recognition, spatial learning, and fear conditioning tasks, while direct injection of FNDC5/irisin into the lateral ventricle of AD mice enhances memory. Moreover, blocking peripheral or cerebral irisin negates the neuroprotective benefits of exercise [205]. Irisin further protects the hippocampus—one of the brain regions most vulnerable to AD—by preventing Aβ accumulation, potentially inhibiting or delaying disease progression [239]. *FNDC5* gene deletion impairs cognitive function in AD patients [245], while peripherally administered irisin crosses the BBB and halts cognitive decline in AD murine models [245].

Beyond Aβ-related mechanisms, irisin exerts neuroprotection through additional pathways disrupted in AD. In vitro studies demonstrate that irisin pre-treatment protects astrocytes from Aβ-induced neurotoxicity by preserving cell viability and reducing pro-inflammatory cytokine release (IL1β, IL6) [246,247]. Furthermore, irisin downregulates the TLR4/MYD88/NF-κB signaling cascade, a key driver of neuroinflammation and neurodegeneration.

As stated before, several studies have shown that administering lactate in murine models can reverse deficits in long-term potentiation, improve learning abilities, and decrease Aβ accumulation in AD models [100,101]. Notably, *TLR4* upregulation and microglial activation have been observed in AD patients and AD-like murine models, contributing to neuronal damage and cognitive impairment [248,249,250]. Additionally, recent studies have identified ADAM10 as the primary α-secretase involved in the cleavage of amyloid-β precursor protein (AβPP) [251,252], and its role in AβPP processing may contribute to AD. Recently, it has been shown that irisin upregulates *ADAM10* expression. Through this mechanism, irisin may exert protective effects on the brain by enhancing α-secretase activity, promoting synaptic health, and potentially reducing amyloid pathology, suggesting a promising link between exercise, molecular signaling, and reduced risk of neurodegeneration [253]. Furthermore, irisin activates AMPK, leading to the inhibition of mTOR and subsequent enhancement of autophagy, facilitating the clearance of amyloid-beta plaques and tau tangles [254]. Irisin, by activating NRF2, inhibiting NF-κB, and enhancing MAPK1/3 signaling, addresses key pathological features of AD, including oxidative stress, inflammation, and neuronal dysfunction. These effects highlight the importance of irisin in maintaining neuronal health and its promising role in AD management [255].

Finally, telomere shortening, a hallmark of aging, is closely associated with AD progression, cognitive decline, Aβ accumulation, and tau phosphorylation. Plasma irisin levels are strongly correlated with telomere length, suggesting potential anti-aging effects and a protective role in AD [256].

### 4.2. Irisin and Parkinson’s Disease

PD is a progressive neurodegenerative disease, pathologically characterized by the loss of dopaminergic neurons, primarily in the substantia nigra, located in the midbrain. It is associated with Lewy bodies, cytoplasmatic deposits containing insoluble aggregates of α-syn [257]. The clinical diagnosis of PD primarily relies on motor symptoms, including a gradually worsening asymmetric resting tremor, cogwheel rigidity, and bradykinesia. However, non-motor symptoms, such as anosmia, constipation, depression, and REM sleep behavior disorder, may emerge years before the onset of motor symptoms. In the later stages of the disease, additional non-motor symptoms, such as autonomic dysfunction, pain, and cognitive decline, can also develop [257].

The development of PD involves neuroinflammation and oxidative stress, driven by inflammatory reactions around neurons, activation of glial cells, and infiltration of immune cells from the periphery [258].

Current treatments for PD, such as dopaminergic supplementation and surgical procedures like deep brain stimulation, mainly aim to alleviate symptoms but have limited long-term effectiveness [259].

Due to these limitations, physical exercise has been recognized as a helpful complementary treatment with potential neuroprotective benefits for the brain. Research has shown that different types of physical exercise can significantly improve PD symptoms, likely by influencing various cytokines and signaling pathways [260].

Increasing lactate availability in the CNS has been shown to be especially effective in slowing the progression of neurodegenerative diseases. However, as mentioned earlier, it is important to note that high levels of lactate could harm brain health, as research suggests that elevated lactate levels worsen symptoms of PD [103]. In this context, clinical studies have shown lower plasma levels of irisin in PD patients, with a negative correlation to disease severity and a positive correlation with cognitive performance ratings [261].

This indicates that lower irisin levels might be associated with the neurodegenerative changes observed in PD. In addition, studies have demonstrated that irisin can improve dopamine uptake in the striatal regions of PD patients, especially in areas opposite the affected limbs, suggesting that irisin may offer neuroprotective benefits by influencing the dopaminergic system [261].

It has been demonstrated that irisin may exert its beneficial effects on PD by alleviating inflammatory stress, modulating autophagy, and maintaining mitochondrial homeostasis [140].

Irisin pre-treatment of microglial cells, cultured in vitro and exposed to α-syn preformed fibrils (α-syn PFFs), reduced the expression and secretion of pro-inflammatory cytokines, including IL6, IL1β, and TNF-α. This was achieved through the suppression of NLRP3 inflammasome activation and its downstream effectors, such as caspase-1 and GSDMD. Irisin also alleviated oxidative stress induced by α-syn PFFs, decreasing malondialdehyde (MDA) levels and restoring SOD and glutathione (GSH) levels, along with mitochondrial membrane potential. The conditioned medium from these cultures, with and without irisin pre-treatment, was used to assess neurotoxicity and mitophagy. Irisin effectively reduced neurotoxicity and promoted mitochondrial clearance and autophagy. In vivo, experiments using a PD mouse model demonstrated that irisin improved motor function by reducing α-syn aggregation, neuroinflammation, and neurodegeneration [262].

In the context of PD, CREB is crucial for the expression of neuroprotective genes, including *NURR1*, which is crucial for the maintenance of dopaminergic neurons. A study demonstrated that inactivation of CREB leads to downregulation of *NURR1*, exacerbating dopaminergic degeneration in a mouse model of PD [263]. Irisin has been shown to upregulate AKT and MAPK1/3, which are upstream of CREB. By modulating these pathways, irisin may indirectly influence CREB activity, thereby supporting neuroprotection in PD [262]. Although direct evidence linking irisin to CREB activation in PD is limited, the convergence of irisin’s effects on upstream signaling pathways with CREB’s role in neuroprotection suggests a potential modulatory relationship.

Further research is needed to clarify the direct interactions between irisin and CREB in PD.

### 4.3. Irisin and Amyotrophic Lateral Sclerosis

Amyotrophic lateral sclerosis (ALS) is a progressive neurodegenerative disease that affects both upper and lower motor neurons. The factors contributing to neuronal damage and cell death remain uncertain, but neuroinflammation seems to play a crucial role in the progression of the disease.

The onset and progression of ALS are driven by the infiltration of activated microglia and astrocytes, which exert a neurotoxic effect through the release of pro-inflammatory cytokines [264,265].

Currently, only a limited number of studies have explored the relationship between irisin and ALS. Irisin has been reported to activate the NRF2 pathway, leading to increased expression of antioxidant enzymes and reduced oxidative stress. Additionally, irisin inhibits NF-κB activation, thereby reducing the production of pro-inflammatory cytokines. These effects suggest that irisin may offer therapeutic potential in ALS by modulating oxidative stress and inflammation pathways [266]. In this regard, indirect evidence has shown that in genetically modified superoxide dismutase type 1 (SOD1) mice, which are the only ALS mouse model currently used, there was an increase in the peripheral canonical PGC1α system, while specific PGC1α isoforms were reduced in the CNS [267]. These findings suggest that changes in the PGC1α system may play a role in the neurodegeneration associated with ALS.

Lactic acid has been shown to stimulate the PGC1α system, creating a direct connection between brain metabolism and neuroprotection. Interestingly, PGC1α is one of the key regulators of *FNDC5* expression [15]. Thus, the link between PGC1α and neurodegeneration in SOD1 mice may be correlated to changes in the expression of *FNDC5*/irisin in either muscle or neurons.

Furthermore, a study evidenced that ALS patients with metabolic alterations had higher serum irisin levels than normo-metabolic ALS patients and healthy controls [268].

To date, there are no studies directly proving the involvement of irisin in ALS. However, this remains an intriguing area of research because ALS patients experience significant muscle damage, and it is known that irisin has anabolic effects on skeletal muscle. Therefore, irisin could serve as a potential biomarker for muscle damage in neurodegenerative diseases.

### 4.4. Irisin and Frontotemporal Dementia

Frontotemporal dementia (FTD) is a subtype of dementia that primarily affects individuals aged 65 or younger. It is characterized by impairments in behavior, language, or executive function. FTD exhibits a wide range of clinical manifestations, and the behavioral variant (bvFTD) is the most prevalent [269].

Chronic low-level inflammation is commonly observed in both the CNS and the bloodstream of patients with FTD [270]. Inflammatory dysregulation may play a significant role in the neurodegeneration and pathological progression of FTD [271].

A study compared the irisin levels between bvFTD patients and healthy controls to investigate a possible correlation between irisin and inflammation [272]. The results highlighted that irisin levels were correlated with the measured pro-inflammatory cytokines. Interestingly, these correlations were not found in the control group. Additionally, some evidence suggests that irisin functions in vivo as a neuroprotective agent, promoting synaptic plasticity and neurogenesis, enhancing memory performance, and regulating the production of BDNF [74,273]. Nevertheless, further studies are needed to deepen the relationship between irisin and FTD.

### 4.5. Irisin and Multiple Sclerosis

Multiple sclerosis (MS) is a chronic condition characterized by neurodegeneration and autoimmune inflammation. It involves the infiltration of autoreactive T and B lymphocytes and macrophages, leading to inflammation at multiple sites. This results in damage to the myelin, axons, and neurons, leading to neuronal loss and gliosis, affecting both the white and gray matter of the CNS [274]. Traditionally, biomarkers for MS diagnosis and prognosis have relied on cerebrospinal fluid analysis [275]. However, irisin has recently emerged as a promising serum biomarker for MS.

In studies involving relapsing-remitting MS (RRMS) patients, significantly lower serum irisin levels have been reported compared to age- and gender-matched controls, with a concomitant increase in the body-mass index (BMI) observed in patients [276]. These findings suggest that reduced irisin levels may contribute to MS pathogenesis through mechanisms such as inflammation, oxidative stress, and apoptosis—processes known to drive demyelination and neuronal loss.

Further investigations into gender-related differences found that both male and female RRMS patients exhibited notably lower irisin concentrations (approximately 45% reduction in males and 35% in females) compared to controls [277]. This reinforces the notion that irisin’s anti-inflammatory and antioxidative properties play a key role in counteracting neurodegenerative processes in MS [74,278].

Adding to its potential as a therapeutic marker, a randomized, prospective study demonstrated that aerobic exercise significantly increased serum irisin levels (Δirisin) and improved aerobic capacity (ΔVO_2_max) in RRMS patients, alongside notable reductions in fatigue and depression [279]. These results imply that exercise-induced elevation of irisin may enhance neuroplasticity and brain function, a hypothesis further supported by evidence of irisin’s antidepressant-like effects through the regulation of energy metabolism in the prefrontal cortex [83].

Collectively, these studies underscore the potential of irisin as both a biomarker and a therapeutic target in MS, offering promising insights for disease monitoring and the development of non-pharmacological interventions.

Table 1 summarizes the key molecular pathways involved in neurodegenerative diseases, highlighting their roles in AD, PD, ALS, FTD, and MS, along with the modulatory effects of irisin on these pathways.

## 5. Potential Applications of Irisin

### 5.1. Irisin as a Biomarker and Therapeutic Target: Potential and Challenges

The potential of irisin (and its precursor FNDC5) as a diagnostic biomarker and therapeutic target has been extensively explored in the last decades [78,318,319], although its role is not yet fully established. Evidence obtained may reveal critical information to fully delineate irisin physiological action and pose the basis for its use in the clinical field. Nevertheless, related technical difficulties may hinder the translation of this knowledge to clinical use. In this section, we focused on irisin potential role as a biomarker in various conditions as well as potential challenges in its use.

### 5.2. Irisin Diagnostic Potential in Metabolic Diseases

Several investigations have focused on FNDC5/irisin levels in the context of pathological conditions [138,320,321,322], especially metabolic disorders [151,323,324,325,326,327,328] with mixed outcomes. Generally, changes in irisin levels have been observed in conditions such as obesity, type 2 diabetes, and insulin resistance [18,329], which support the use of irisin as a potential indicator of metabolic decline. Indeed, this myokine may reflect early metabolic derangement before traditional markers show changes [330].

Of particular interest, the genetic profiling of the FNDC5 gene for mutations may be used as a very early indicator of metabolic dysfunction. Indeed, genetic variations in the *FNDC5* gene have been linked to differences in insulin sensitivity, lipid profiles, and BMI across diverse populations [71,72,331,332,333]. These associations suggest that irisin may serve as a valuable biomarker for metabolic health, helping not only to monitor the efficacy of lifestyle interventions but also to predict the risk of developing metabolic disorders based on the related genetic profile.

### 5.3. Challenges in Irisin Detection and Standardization

Despite the promising data, the use of irisin as a biomarker presents several challenges. Many studies have shown inconsistent or mixed results, questioning its use as a biomarker [151,323,324,325,326,328]. Notably, early studies relied heavily on antibody-based detection methods (ELISA, Western blot), which have been criticized due to their lack of specificity [40,41,163,334]. A possible alternative could be the use of mass spectrometry, which offers greater reliability [84]. However, this method still requires standardized protocols and reference ranges to obtain accurate measurements of irisin before its use in clinical settings. These technical challenges, along with the limited knowledge regarding the exact mechanisms involved in its action, should be taken into consideration to fully exploit the potential of irisin as a biomarker. This highlights the need for further studies to better define the role of this myokine.

## 6. Conclusions: Present and Future Perspectives

The studies discussed throughout this review consistently highlight the multifaceted neuroprotective potential of irisin. Evidence shows that this exercise-induced myokine not only mitigates neuroinflammation by reducing microglial and astrocyte activation and suppressing the release of proinflammatory cytokines, but also counteracts oxidative stress, improves mitochondrial function, and preserves BBB integrity. Through these actions, irisin helps prevent neuronal damage, synaptic loss, and the accumulation of misfolded proteins—key contributors to neurodegenerative disorders such as AD, PD, ALS, FTD, and MS.

Moreover, irisin appears to mimic several beneficial effects of physical exercise by upregulating neurotrophins such as BDNF, which is essential for neuronal survival, synaptic plasticity, and cognitive functions. Its capacity to enhance insulin signaling and modulate metabolic homeostasis further supports its potential role in preventing insulin resistance often associated with neurodegenerative conditions.

Looking toward future applications, the therapeutic potential of irisin is compelling. Recombinant irisin administration has been shown to reproduce many of the beneficial effects of exercise in preclinical models, suggesting its potential as a novel treatment strategy. However, several challenges remain. Key aspects of irisin’s mechanism of action—such as the complete characterization of its receptors, the regulation of FNDC5 cleavage, and the interplay with ncRNAs—require further investigation. In addition, technical challenges related to its detection and standardization must be overcome before irisin can be reliably employed as a clinical biomarker or therapeutic agent.

In summary, while current findings highlight irisin’s capacity to protect the CNS from various insults, additional research is needed to fully harness its therapeutic potential. Until then, these insights emphasize the importance of physical exercise as a natural, accessible strategy for maintaining neurological health, particularly in aging and sedentary populations. Future studies will be pivotal in translating the promising neuroprotective properties of irisin into effective clinical interventions for neurodegenerative diseases.

## Figures and Tables

**Table 1:** Overview of the main molecular pathways implicated in major neurodegenerative diseases (AD, PD, ALS, FD, and MS), and the modulatory effects of irisin on these processes. The table highlights how irisin influences key pathways involved in neuroprotection, antioxidant defense, inflammation modulation, and cellular homeostasis across different neurological disorders.

Pathway	Role in AD	Role in PD	Role in ALS	Role in FTD	Role in MS	Effect of Irisin
NRF2	Regulates antioxidant defense; reduced activity linked to Aβ accumulation [280]	Protects dopaminergic neurons from oxidative stress [281]	Supports antioxidant gene expression; impaired in ALS [282]	May protect against oxidative damage in neurons [283]	Reduces oxidative stress in oligodendrocytes [284]	Activates NRF2; increases antioxidant enzymes (e.g., SOD, HO1) [285]
NF-κB	Promotes neuroinflammation; activated by Aβ and tau [286]	Involved in dopaminergic neuron inflammation [287]	Chronic activation linked to glial inflammation [288]	Associated with TDP-43-mediated inflammation [289]	Drives demyelination and T-cell activation [290]	Inhibits NF-κB activation; reduces pro-inflammatory cytokines (e.g., IL1β, TNFα) [291]
PI3K/AKT	Supports neuronal survival; impaired in AD [292]	Protects neurons; promotes dopamine synthesis [293]	Promotes motor neuron survival [294]	Regulates neuronal viability [295]	Maintains BBB and modulates immune response [296]	Activates AKT; promotes cell survival and neurogenesis [297]
AMPK	Regulates autophagy; dysfunction linked to tau pathology [298]	Controls mitochondrial biogenesis and autophagy [299]	Maintains energy balance and autophagy in neurons [300]	Modulates metabolism and neuroinflammation [300]	Regulates immune cell energy status and reduces inflammation [301]	Activates AMPK; improves mitochondrial function and autophagy [187]
BDNF/CREB	Decreased BDNF levels in AD [302]	Supports dopaminergic neuron survival and plasticity [303]	May maintain neuromuscular synapse function [304]	Reduces synaptic loss and supports behavioral flexibility [305]	Enhances remyelination and synaptic integrity [306]	Increases BDNF expression via CREB; promotes synaptic plasticity [307]
JAK/STAT	Involved in cytokine-mediated neuroinflammation [308]	Modulates glial activity and neuron-glia communication [309]	Drives inflammatory glial activation in ALS [310]	Linked to neuroinflammatory signaling [311]	Critical in autoimmune demyelination [312]	May inhibit excessive cytokine production; modulates immune signaling pathways [21]
Autophagy (mTOR, PINK1/Parkin)	Impaired clearance of Aβ and tau; contributes to AD pathology [313]	Dysfunction leads to α-syn aggregation [314]	Downregulated in ALS murine models. Crucial for clearance of protein aggregates and damaged mitochondria [315]	Deficits linked to TDP-43 accumulation, which is involved in FTD etiopathogenesis [316]	Enhances myelin repair by clearing damaged organelles [317]	Activates autophagy via AMPK; promotes clearance of protein aggregates [262]

## Abbreviations

The following abbreviations are used in this manuscript:

α-syn	α-synuclein
AβPP	amyloid-β precursor protein
ACSL4	Acyl-CoA Synthetase Long-Chain Family Member 4
AD	Alzheimer’s Disease
ADAM10	ADAM Metallopeptidase Domain 10
AKT	AKT Serine/Threonine Kinase
ALS	Amyotrophic lateral sclerosis
AMPK	AMP-activated protein kinase
ATF4	Activating Transcription Factor 4
Aβ	amyloid beta
BAX	BCL2 Associated X, Apoptosis Regulator
BBB	Blood-Brain Barrier
BCL2	BCL2 Apoptosis Regulator
BDNF	Brain Derived Neurotrophic Factor
BMI	body-mass index
bvFTD	behavioral variant FTD
cAMP	Cyclic adenosine monophosphate
CASP3/9	Caspase 3/9
CAV1	caveolin 1
CMA	chaperone-mediated autophagy
CNS	Central Nervous System
CREB	cAMP response element-binding protein
DDIT3	DNA Damage Inducible Transcript 3
DG	dentate gyrus
DOAJ	Directory of open access journals
EIF2A	Eukaryotic Translation Initiation Factor 2A
FAK	focal adhesion kinase
FNDC5	fibronectin type III domain-containing protein 5
FNIII	fibronectin type III
FOXO1	Forkhead box protein O1
FTD	Frontotemporal dementia
G6Pase	Glucose-6-Phosphatase
GPX4	Glutathione Peroxidase 4
GSDMD	Gasdermin D
GSH	glutathione
GSK3	Glycogen Synthase Kinase 3
GYS1	Glycogen synthase
HIF1α	hypoxia-inducible factor 1 subunit α
HMOX1	heme oxygenase-1
Hsp90α	Heat Shock Protein 90 Alpha
IGF1	Insulin Like Growth Factor 1
IGF1R	Insulin Like Growth Factor 1 Receptor
IL1β	Interleukin 1 Beta
IL6	Interleukin 6
iNOS	inducible nitric oxide synthase
IRF3	interferon regulatory
IRS1	Insulin Receptor Substrate 1
IRS2	Insulin Receptor Substrate 2
IκB	Inhibitor of Nuclear Factor Kappa B Kinase
JAK2	Janus Kinase 2
LDH	lactate dehydrogenase
LD	Linear dichroism
LPS	lipopolysaccharide
MAP1LC3B	Microtubule Associated Protein 1 Light Chain 3 Beta
MAPK	Mitogen-Activated Protein Kinase
MAPK1 or ERK2	Mitogen-Activated Protein Kinase 1
MAPK14 or p38	Mitogen-Activated Protein Kinase 14
MAPK3 or ERK1	Mitogen-Activated Protein Kinase 3
MAPK8	Mitogen-Activated Protein Kinase 8
MBA	muscle-brain axis
MCT2	monocarboxylate transporter 2
MDA	malondialdehyde
MDPI	Multidisciplinary Digital Publishing Institute
MFN2	Mitofusin 2
MS	Multiple sclerosis
MTOR	Mechanistic Target of Rapamycin Kinase
MT-CO2	cyclooxygenase 2
MYD88	MYD88 Innate Immune Signal Transduction Adaptor
NCD	non-communicable disease
ncRNA	Non-coding RNA
NF-κB	nuclear factor kappa-light-chain-enhancer of activated B cells
NGF	nerve growth factor
NICD	Active Notch intracellular domain
NLRP3	NLR Family Pyrin Domain Containing 3
NQO1	NAD(P)H Quinone Dehydrogenase 1
NRF1	NFE1 Like BZIP Transcription Factor 1
NRF2 or NFE2L2	NFE2 Like BZIP Transcription Factor 2
NTRK2	Neurotrophic Receptor Tyrosine Kinase 2
NURR1	nuclear receptor related 1 protein
OPA1	OPA1 Mitochondrial Dynamin Like GTPase
PCK	Phosphoenolpyruvate Carboxykinase
PD	Parkinson’s disease
PFFs	Preformed fibrils
PGC1α	peroxisome proliferator-activated receptor gamma coactivator 1-alpha
PI3K	Phosphatidylinositol-4,5-Bisphosphate 3-Kinase
PINK1	PTEN Induced Kinase 1
PKA	Protein Kinase cAMP-Activated
POCD	Postoperative cognitive dysfunction
PPARγ	Peroxisome Proliferator Activated Receptor Gamma
PRKN	Parkin
ROS	reactive oxygen species
RRMS	relapsing-remitting MS
SIRT1	Sirtuin 1
SLC2A or GLUT4	Solute Carrier Family 2 (Facilitated Glucose Transporter), Member 4
SMAD3	SMAD Family Member 3
SNAI1	Snail Family Transcriptional Repressor 1
SOD	superoxide dismutase
SOD1	superoxide dismutase type 1
SQSTM1	Sequestosome 1
SREBF2	Sterol Regulatory Element Binding Transcription Factor 2
STAT3	Signal Transducer and Activator of Transcription 3
STAT6	Signal Transducer and Activator of Transcription 6
TCA	tricarboxylic acid cycle
TFAM	Transcription Factor A, Mitochondrial
TGFβ	Transforming growth factor β
TLA	Three letter acronym
TLR4	Toll-like receptor 4
TNFα	Tumor Necrosis Factor
UCP1	uncoupling protein 1
ULK1	Unc-51 Like Autophagy Activating Kinase 1
VEGF	vascular endothelial growth factor
VO_2_max	maximal oxygen uptake
